# Astrocyte elevated gene-1 promotes tumour growth and invasion by inducing EMT in oral squamous cell carcinoma

**DOI:** 10.1038/s41598-017-15805-8

**Published:** 2017-11-13

**Authors:** Yan Wang, Ting Wang, Yunduan Sun, Wenjing Sun, Xiumei Wang

**Affiliations:** 1 Department of Dentistry, the Second Affiliated Hospital of Harbin Medical University, Harbin, 150086 China; 2 Department of Ophthalmology, the First Affiliated Hospital of Harbin Medical University, Harbin, 150001 China; 3 Laboratory of Medical Genetics, Harbin MedicalUniversity, Harbin, 150081 China

## Abstract

Oral squamous cell carcinoma (OSCC) is a common human malignancy with a high incidence rate and poor prognosis. Although astrocyte elevated gene 1 (AEG-1) expression is up-regulated in various human cancers and plays an important role in carcinogenesis and tumour progression, the impact of AEG-1 on the development and progression of OSCC remains unclear. Accordingly, this study aims to clarify the biological significance of AEG-1 in OSCC. We found AEG-1 to be overexpressed in OSCC tissues compared to normal oral mucosa. Knockdown or overexpression of AEG-1 in OSCC cell lines showed that AEG-1 is important for tumour growth, apoptosis, drug tolerance, and maintaining epithelial-mesenchymal transition (EMT)-mediated cell migration and invasion *in vitro*. Moreover, in a xenograft-mouse model generated by AEG-1-overexpressing SCC15 cells, we found that higher expression of AEG-1 promoted tumour growth, angiogenesis, and EMT *in vivo*. These findings provide mechanistic insight into the role of AEG-1 in regulating OSCC tumour growth, apoptosis, drug tolerance, and invasion, as well as AEG-1-induced activation of p38 and NF-κB signalling, suggesting that AEG-1 is an important prognostic factor and therapeutic target for OSCC.

## Introduction

Head and neck squamous cell carcinoma (HNSCC) is the sixth most common cancer worldwide. Each year, 1.6 million new cases of HNSCC are diagnosed, with half localized in the oral cavity (oral squamous cell carcinoma, OSCC), and 33,3000 deaths^1^. Indeed, OSCC is an important component of the worldwide cancer burden; despite radical surgery combined with radiation, chemotherapy and targeted therapy, OSCC has a 5-year survival rate of only approximately 50%^2^. The pathogenesis of OSCC is complex, involving many genes and pathways, though the mechanism of OSCC development remains unclear.

Metastasis is dependent on unique mechanisms by which cancer cells escape from the primary tissue and spread to surrounding tissues. As part of the epithelial-mesenchymal transition (EMT), molecular reprogramming is a crucial step in the metastasis of most carcinomas^3^. During metastatic progression, EMT causes primary epithelial-like tumour cells to acquire invasive potential, including increased motility and mesenchymal characteristics, which results in dissemination from the original tumour and intravasation into the tumour vessel. EMT-driven cells circulating in the blood then redifferentiate into a primary status *via* the mesenchymal-epithelial transition (MET) during colonisation of and growth at distant metastatic sites^4,5^. Because of the important roles of EMT in the metastatic process, controlling EMT progression in tumours is thought to be a promising strategy to inhibit metastasis and prolong patient survival.

Astrocyte elevated gene-1 (AEG-1), also known as metadherin (MTDH) or LYsine-RIch CEACAM1 co-isolated (LYRIC), is a 582-amino acid, type II transmembrane protein without any known functional domains. Recently, numerous reports have demonstrated that AEG-1 might play a pivotal role in the pathogenesis, progression, invasion, metastasis and overall patient survival of multiple human cancers^6–14^. AEG-1 overexpression in human breast cancer cells and HEK293T cells increases metastasis *in vivo*, especially to the lungs, whereas inhibition of AEG-1 expression decreases cell migration and invasion^15^. Furthermore, overexpression of AEG-1 in HCC cells increases the production of angiogenic factors, such as vascular endothelial growth factor (VEGF), placental growth factor, and fibroblast growth factor α^11^. In addition to its implication as an oncogene participating in pathways critical to HNSCC^16^, overexpression of AEG-1 in OSCC appears to be associated with a more aggressive disease, poorer prognosis and shorter survival^17^. Regardless, the role of AEG-1 in mediating OSCC metastasis has not been well investigated to date.

Here, we report that AEG-1 is overexpressed in most OSCC clinical specimens. Using gain-or loss-of-function assays, we validated that AEG-1 can promote aggressive behaviour in OSCC cell lines both *in vitro* and *in vivo*. AEG-1 was also found to contribute to EMT-mediated metastasis in OSCC.

## Results

### AEG-1 is overexpressed in OSCC

**Figure 1:**
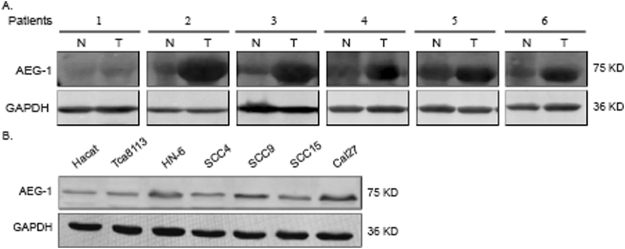
The AEG-1 protein level in tissue specimens and OSCC cells is illustrated. (**A**) The AEG-1 protein level was determined in six paired tissue specimens from patients with OSCC using immunoblotting analyses. The images were obtained from different parts of the same gel; GAPDH was detected as a control. (**B**) Cell extracts were prepared from Hacat, Tca8113, HN-6, SCC4, SCC9, SCC15, CAL-27 cells and analysed by immunoblotting analyses with an anti-AEG-1 antibody. The images were obtained from different parts of the same gel; GAPDH was detected as a control.

We detected AEG-1 expression in OSCC tissue specimens using immunoblotting analysis. Compared to controls, AEG-1 protein expression levels in six OSCC tissues were increased, most obviously in patient 2, patient 3 and patient 6 (Fig. 1A). We also investigated expression of AEG-1 in six OSCC cell lines (Tca8113, HN-6, SCC4, SCC9, SCC15, and CAL-27) by immunoblotting, with the human skin keratinocyte cell line Hacat used as a control (Fig. 1B). AEG-1 expression in cell lines HN-6, SCC9, and CAL-27 was higher than that in Hacat cells, and AEG-1 was more highly expressed in HN-6, SCC9, and CAL-27 cells than in Tca8113, SCC4 and SCC15 cells. Therefore, we used cell lines Tca8113 and SCC15 in subsequent AEG-1-overexpression studies and SCC9 and CAL-27 in AEG-1-knockdown studies.

### Overexpression of AEG-1 increases cell proliferation and suppression of AEG-1 reduces cell proliferation in OSCC cells *in vitro*

**Figure 2:**
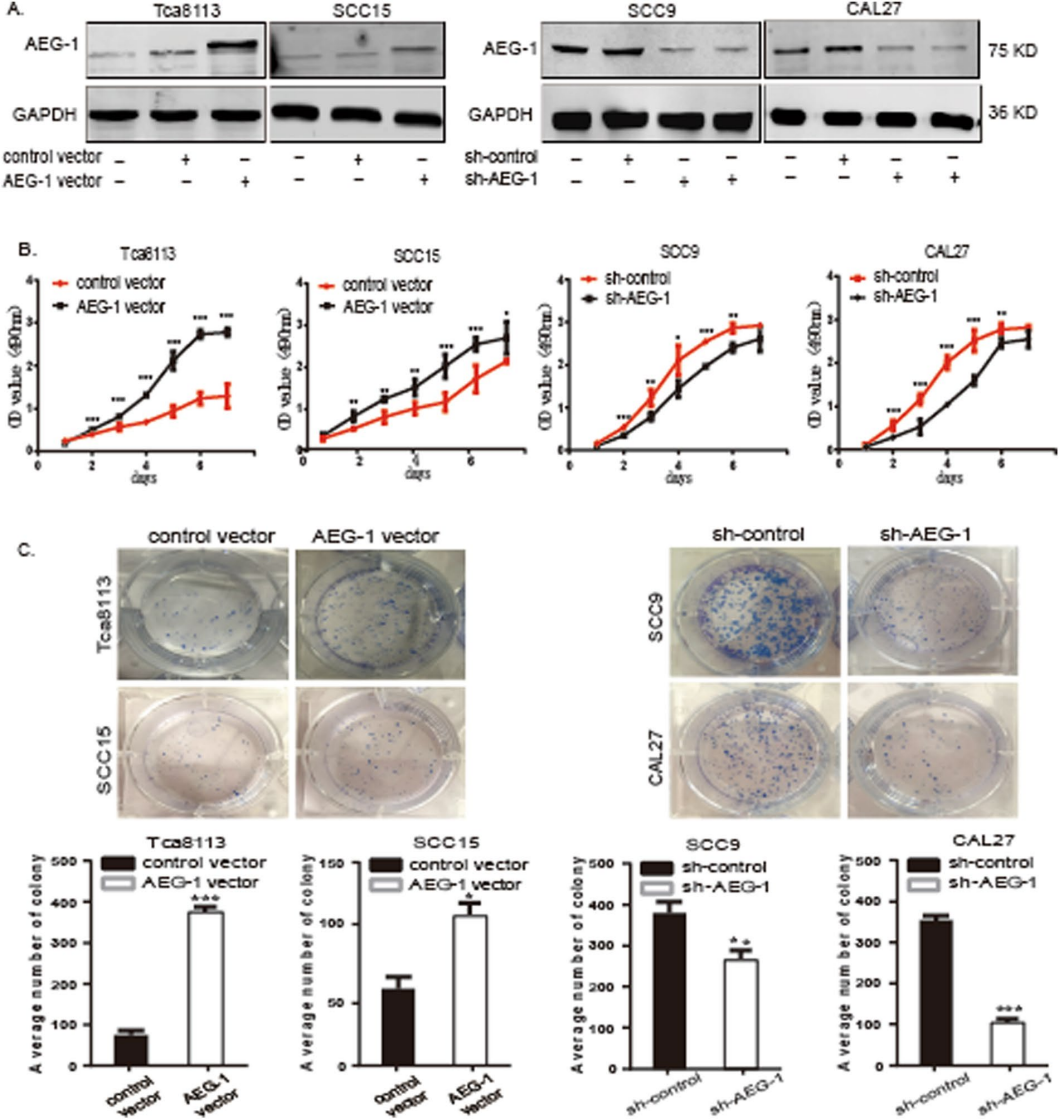
Overexpression of AEG-1 increased the cell growth rate in OSCC cells *in vitro*; knockdown of AEG-1 decreased cell growth. (**A**) Expression levels of AEG-1 were increased/decreased in stable AEG-1-overexpressing/knocked down OSCC cell lines. Protein levels of AEG-1 were detected in stably overexpressing Tca8113 and SCC15 cells and knocked down SCC9 and CAL-27 cells by immunoblotting. The images were obtained from different parts of the same gel; GAPDH was detected as a control. (**B**) The growth rates of AEG-1-overexpressing Tca8113 and SCC15 cells were increased compared to control cells based on the MTS assay. The growth rates of sh-AEG-1 trasnfected SCC9 and CAL-27 cells were decreased compared to control cells based on the MTS assay. The OD value of the cells was measured every day for 7 d and plotted for the mean ± SD. *Indicates *P* < 0.05, **indicates *P* < 0.01, ***indicates *P* < 0.001 by ANOVA. (SD, standard deviation). (**C**) Based on a colony formation assay, stable overexpression of AEG-1 increased cell proliferation in AEG-1 Tca8113 and SCC15 cells, and stable knockdown of AEG-1 decreased cell proliferation in sh-AEG-1 transfected SCC9 and CAL-27 cells. Quantification analyses for C; the data are the mean ± SD of colony numbers. *Indicates *P* < 0.05, **indicates *P* < 0.01, ***indicates *P* < 0.001 by t-tests.

To further investigate the role of AEG-1 in OSCC, we performed the following functional study of AEG-1 in Tca8113, SCC15, SCC9 and CAL-27 cells: we generated stable Tca8113 and SCC15 cell lines transfected with lentivirus for overexpressing AEG-1 and used SCC9 and CAL-27 cell lines with an RNA interference (RNAi) system to knockdown AEG-1. Based on immunoblotting analyses, AEG-1 expression was increased in AEG-1-vector-transfected Tca8113 and SCC15 cells and decreased in sh-AEG-1 (sh-AEG-1-1 and sh-AEG-1-2) SCC9 and CAL-27 cells compared to that in the respective control cells (Fig. 2A). Thus, we used these stable cell lines for further study and selected the best effect from two small hairpin RNA (shRNA) sequences for subsequent experiments. We first examined the cell proliferation ability of the stably transfected cells. The growth rate of AEG-1-overexpressing cells was markedly faster than that of control cells, whereas knockdown cells exhibited a slower growth rate than sh-control cells, with a statistically significant difference from the second day (*P* < 0.05, Fig. 2B).

In addition, we performed a colony formation assay to further illustrate cell proliferation ability. Two thousand AEG-1-vector-transfected Tca8113 and SCC15 cells, control-vector-transfected Tca8113 and SCC15 cells, sh-AEG-1 SCC9 and CAL-27 cells, and sh-control SCC9 and CAL-27 cells were seeded in 6-well plates and photographed after 14 days of growth (Fig. 2C). By scoring the number of colonies for each group, and we found a significantly higher number of colonies for the AEG-1-overexpressing cells than the control cells (*P* < 0.05). Moreover, the colony numbers of the AEG-1-knockdown cells were significantly fewer than those of the sh-control cells in both cell lines (*P* < 0.01). These results indicate that AEG-1 enhanced the tumour proliferation ability of OSCC cells.

### Down-regulation of AEG-1 induces cell cycle arrest in the G_0_/G_1_ phase

**Figure 3:**
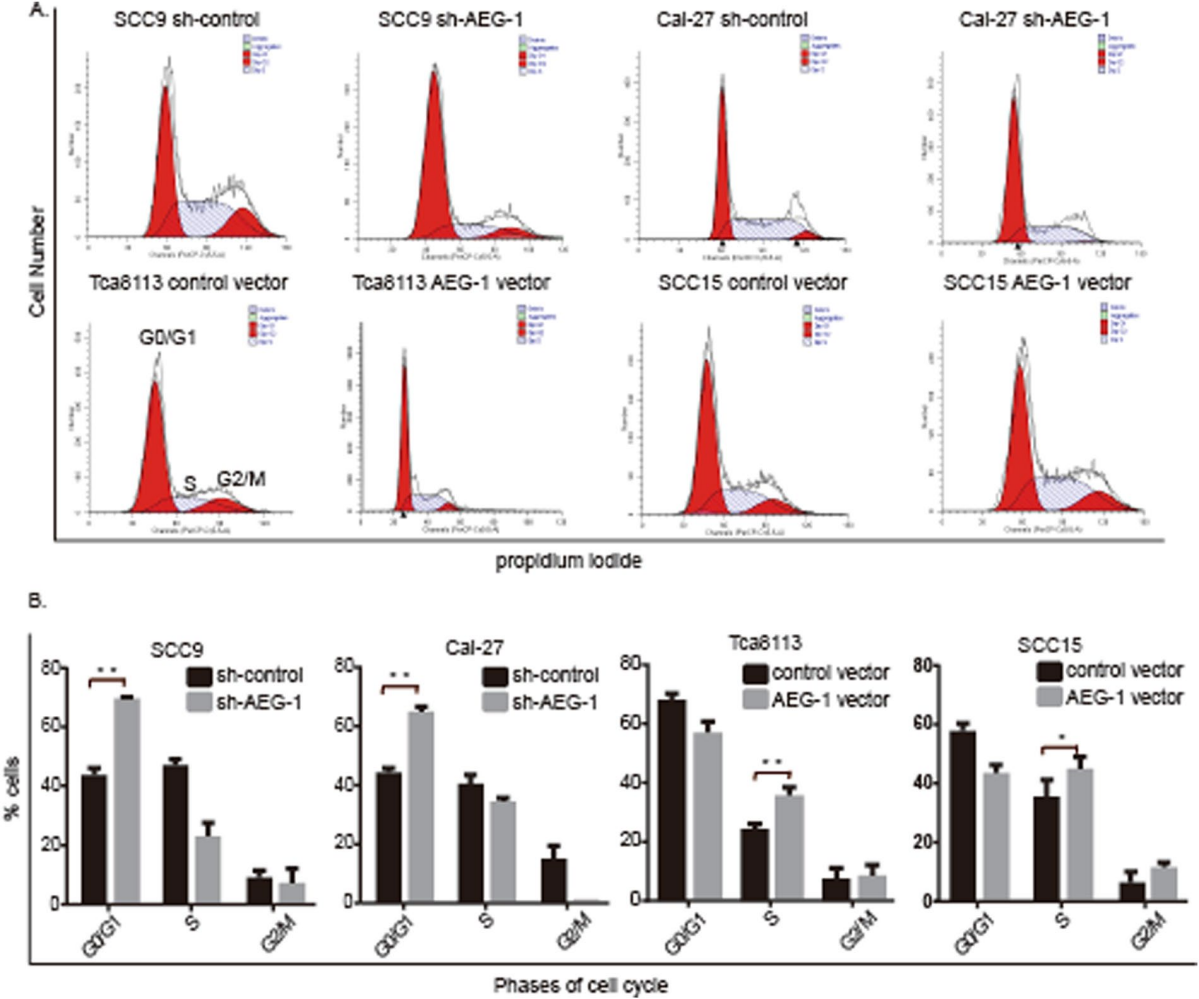
The influence of AEG-1 on cell cycle arrest in OSCC cell lines. (**A**) Down-regulation of AEG-1 induced cell cycle arrest in G_0_/G_1_ phase. The cell cycle in OSCC cells was analysed by flow cytometry. (**B**) Cell cycle data for B. *Indicates *P* < 0.05, **indicates *P* < 0.01.

To further investigate whether the AEG-1 gene influences cell growth *via* cell cycle arrest, propidium iodide staining and flow cytometry for cell cycle analysis were conducted in OSCC cells after sh-AEG-1 vector transfection. We found that down-regulation of AEG-1 led to a typical G_0_/G_1_ arrest pattern in both SCC9 and CAL-27 cells (Fig. 3A,B). Specifically, the percentage of SCC9 cells in G_0_/G_1_ was increased from 41.22% in control-siRNA-treated cells to 69.45% in AEG-1-siRNA-treated cells (Fig. 3B). Additionally, the percentage of CAL-27 cells in G_0_/G_1_ was increased from 46.00% in control-siRNA-treated cells to 63.70% in AEG-1-siRNA-treated cells (Fig. 3B). Therefore, our data suggest that AEG-1-siRNA treatment caused G_0_/G_1_ phase arrest in OSCC cells.

When the AEG-1 gene was overexpressed in Tca8113 and SCC15 cells, the percentage of cells in S and G_2_/M phases was profoundly increased compared to that of control-treated cells for both cell lines. Compared to 34.46% in overexpressing cells of the Tca8113 line, the percentage of cells in S phase was 22.86% in the control group, with a statistically significant difference (*P* < 0.05). Similarly, the percentage of cells in S phase was elevated in SCC15 cells to approximately 31% in the control group to 40% in the overexpressing group (Fig. 3A,B).

### AEG-1 induces CDDP tolerance in OSCC cell lines

**Figure 4:**
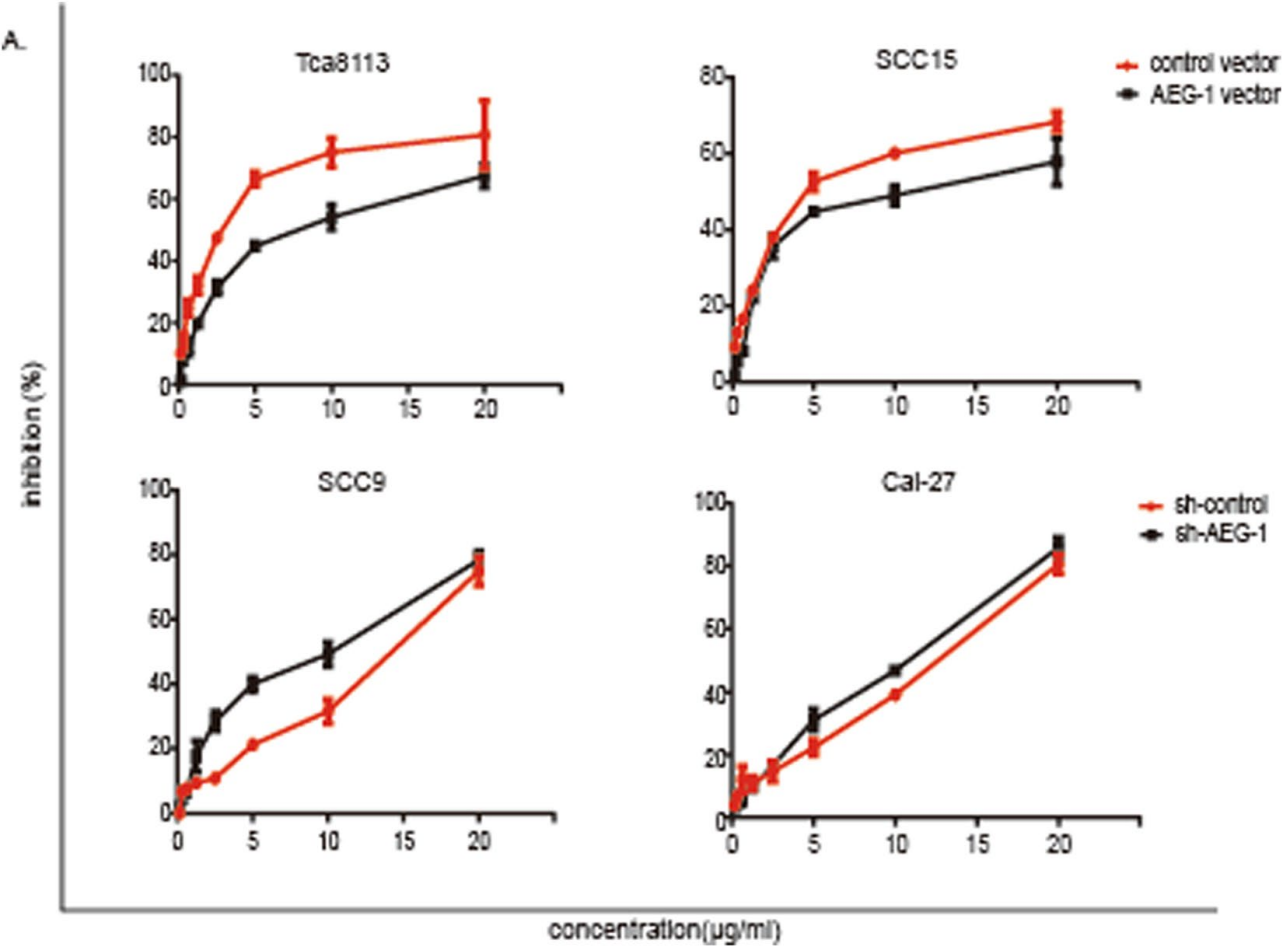
The influence of AEG-1 on chemotherapy resistance in OSCC cell lines. (**A**) All tumour cells showed decreased viability with increasing dose of CDDP, as determined by the MTS assay. The results represent the mean ± SD of three independent experiments.

To investigate the effect of AEG-1 on the viability and drug tolerance of OSCC cell lines, we used an MTS [3-(4,5-dimethylthiazol-2-yl)-5-(3-carboxymethoxyphenyl)-2-(4-sulphophenyl)-2*H*-tetrazolium] dye reduction assay. Cells were treated with increasing concentrations of *cis*-diamminedichloridoplatinum(II) (CDDP) for 72 h, along with the vehicle control (dimethyl sulphoxide, DMSO, 0.05%). All tumour cells exhibited decreased viability with increasing CDDP dose (Fig. 4A). The value at which 50% of growth inhibition was achieved was taken as the IC50 dosage of the extract for the respective treated cell line (Table 1), and values of 2.2, 5.9, 11.3 and 11.1 μg/ml for Tca8113, SCC15, SCC9 and CAL-27 cells, respectively, were obtained at 72 h. The IC50 dosage was decreased in sh-AEG-1 cells compared to that in sh-control cells, whereas AEG-1 overexpression increased the IC50 dosage in Tca8113 and SCC15 cells. These results show that CDDP suppressed cell viability in a dose-dependent manner in OSCC cells and, conversely, that AEG-1 can induce CDDP tolerance in OSCC cell lines.

**Table 1:** IC50 dosage of the extract for the respective OSCC-treated cell line.

Cells	Tca8113		SCC15		SCC9		CAL-27	
	Control vector	AEG-1 vector	Control vector	AEG-1 vector	sh-control	sh-AEG-1	sh-control	sh-AEG-1
IC50 (μg/ml)	2.2	7.3	5.9	8.6	11.3	6.23	11.1	8.8

### AEG-1 promotes tumour migration and invasion of OSCC cells *in vitro*

**Figure 5:**
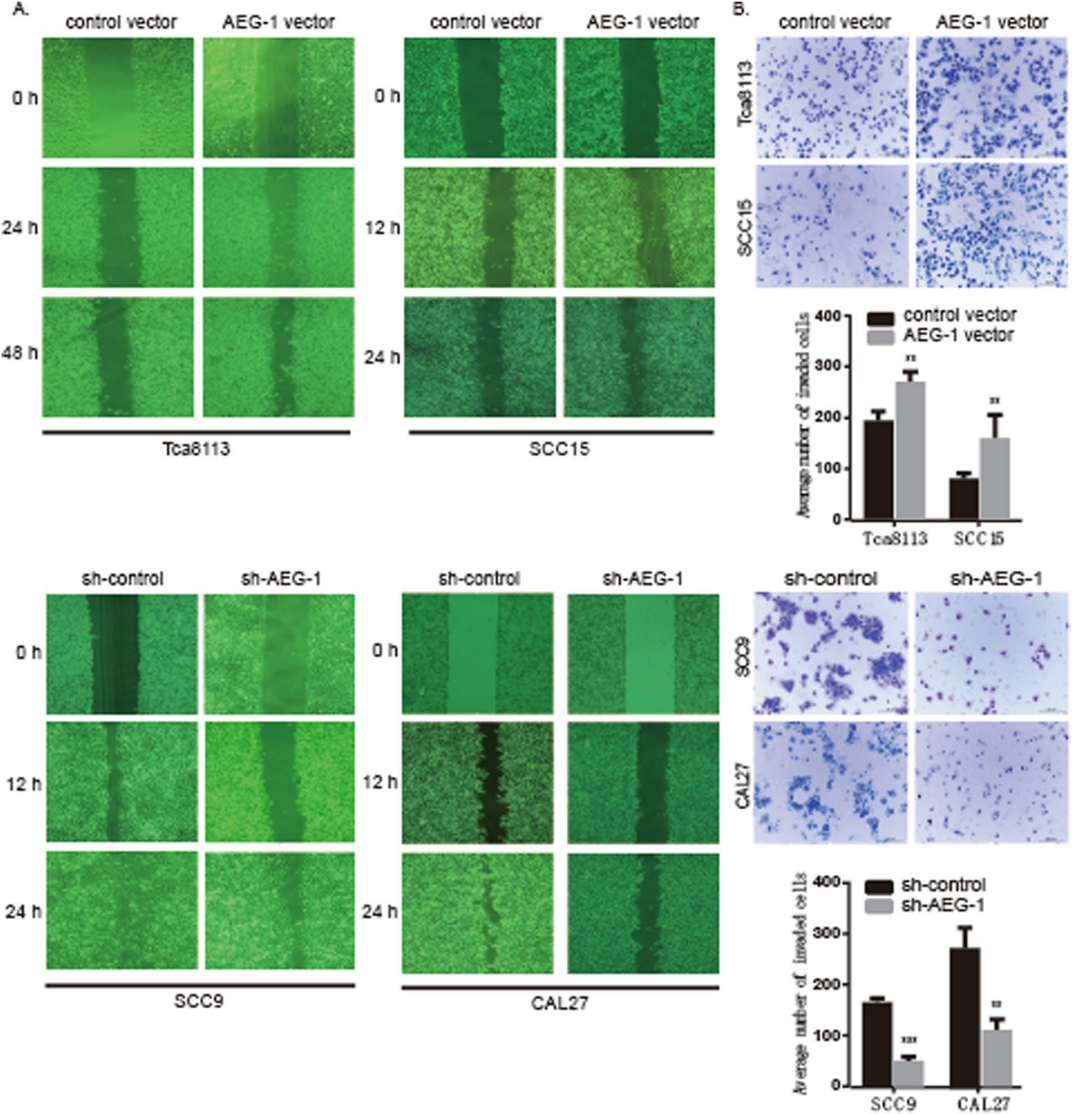
Up-regulation of AEG-1 promoted cell migration and metastasis *in vitro*, whereas knockdown of AEG-1 decreased cell migration and metastasis. (**A**) Up-regulation of AEG-1 promoted migration in Tca8113 and SCC15 cells in a wound-healing assay; knockdown of AEG-1 decreased SCC9 and CAL-27 cell migration in a wound-healing assay. (**B**) Up-regulation of AEG-1 promoted Tca8113 and SCC15 cell invasion in a cell invasion assay; knockdown of AEG-1 decreased invasion in sh-AEG-1-transfected SCC9 and CAL-27 cells in a cell invasion assay compared to sh-control cells, 100× magnifications. Statistical analyses for B; data are the mean ± SD of invading cells. **Indicates *P* < 0.01 by *t*-tests. (SD, standard deviation).

We performed a wound-healing assay to investigate the effects of AEG-1 on OSCC cell migration. At 48 h for Tca8113 cells and 24 h for SCC15 cells, the migration rates of AEG-1-overexpressing cells were higher than those of sh-control cells (Fig. 5A). AEG-1 knockdown inhibited CAL-27 and SCC9 cell migration compared with that of sh-control cells (Fig. 5A). Overall, quantification of the wound healing results suggested that AEG-1 promotes cell migratory capacity. As metastatic ability is the most aggressive function of cancer cells, we examined the metastasis of OSCC cells after transfection using a cell invasion assay. After 48 h of incubation in invasion chambers, more AEG-1-overexpressing cells than control cells invaded through the basement membrane; in contrast, much fewer sh-AEG-1 cells than sh-control cells migrated (*P* < 0.01, Fig. 5B). Using numerical scoring, histograms showed that up-regulation of AEG-1 led to a significantly higher invasion rate in both cell lines, whereas knocking down AEG-1 resulted in lower invasion rates *in vitro*.

### Overexpression of AEG-1 increases OSCC EMT and inhibition of AEG-1 suppresses OSCC EMT *in vitro*

**Figure 6:**
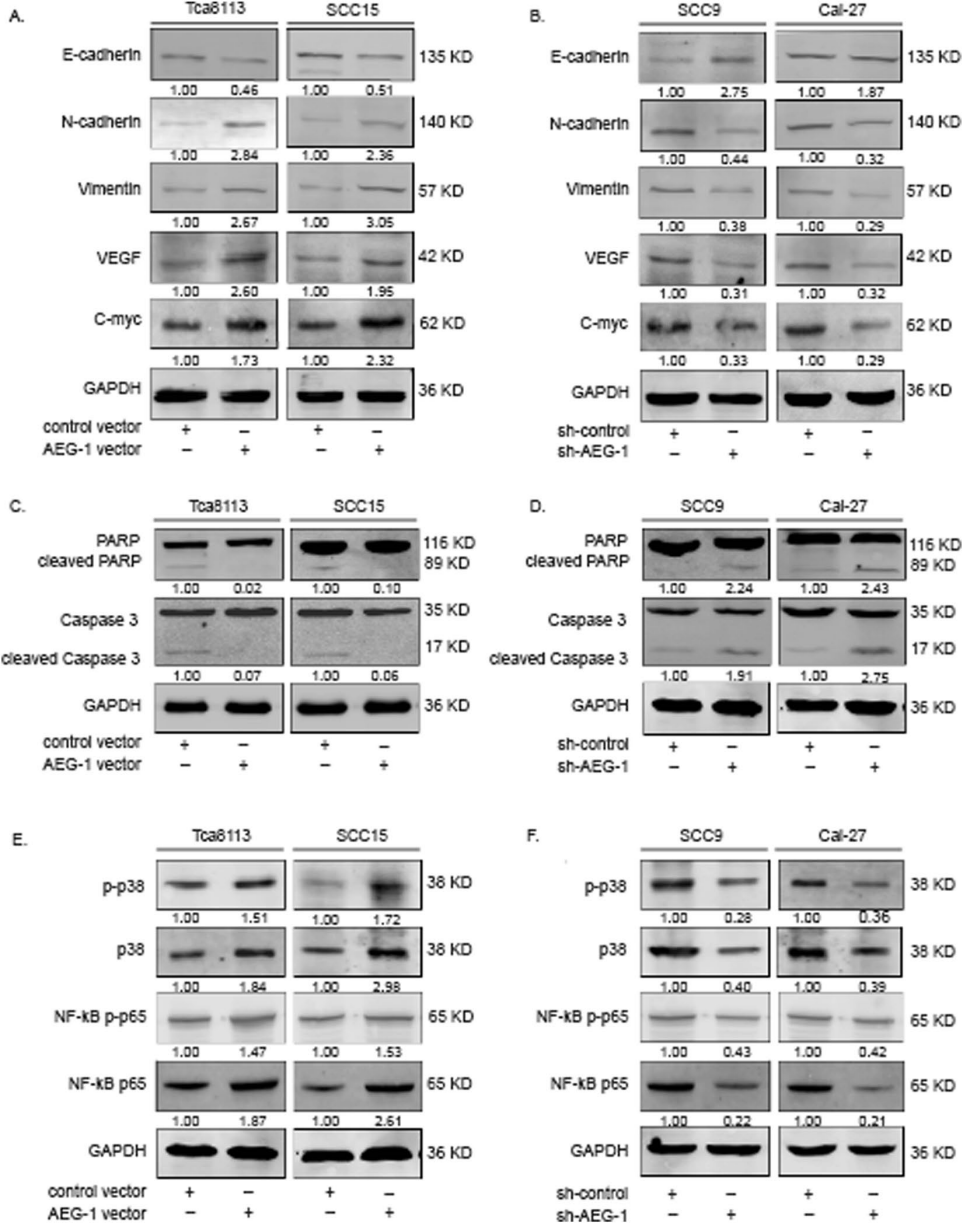
(**A**,**B**) Overexpression of AEG-1 increased OSCC EMT and angiogenesis *in vitro*, whereas inhibition of AEG-1 suppressed OSCC EMT, angiogenesis and progression. (**C**,**D**) Overexpression of AEG-1 suppressed apoptosis in OSCC cells *in vitro*, and inhibition of AEG-1 increased apoptosis in OSCC cells. (**E**,**F**) AEG-1 activated p38 and NF-κB signalling in OSCC cells, as determined by immunoblotting analyses. GAPDH was detected as a control. The images were obtained from different gels, but the experimental condition were the same.

Previous studies have established that EMT is required for metastasis in multiple human epithelial cancers. Consequently, we used western blotting of EMT markers to evaluate whether AEG-1 can promote EMT-mediated OSCC invasion. Specifically, loss-of-function of E-cadherin is believed to initiate EMT and human cancer metastasis^18^. We found that up-regulation of AEG-1 in Tca8113 and SCC15 cells reduced expression of E-cadherin and increased that of N-cadherin and vimentin compared to the negative control group (Fig. 6A). Furthermore, in SCC9 and CAL-27 cells, AEG-1 silencing induced expression of E-cadherin and suppressed that of N-cadherin and vimentin compared to negative control cells (Fig. 6B). We also measured VEGF expression and found that AEG-1 up-regulation or knockdown with increased or decreased expression of VEGF (Fig. 6A,B).

In addition, we checked the oncogene c-Myc expression level with AEG-1 overexpression or kncokdown in OSCC cells. We found c-Myc expression increased in AEG-1 overexpression Tca8113 and SCC15 cells, whereas c-Myc expression reduced in AEG-1 kncokdown SCC9 and CAL-27 cells (Fig. 6A,B). Taken together, these data indicate that AEG-1 can dramatically affect EMT and promote tumour angiogenesis and progression in OSCC cells *in vitro*.

### AEG-1 reduces apoptosis in OSCC cells

We then investigated the effect of AEG-1 on OSSC apoptosis by western blotting analysis. Due to the lack of Caspase-3 and poly(ADP-ribose) polymerase (PARP) cleavage and because tumour cells themselves are resistant to apoptosis, we treated OSCC cells with CDDP (IC50 dosage) for 48 h to induce apoptosis. The results confirmed that CDDP pretreatment significantly enhanced the cleavage of Caspase-3 and PARP in Tca8113, SCC15, SCC9 and CAL-27 cells (Fig. 6C,D). Of note, up-regulation of AEG-1 decreased levels of cleaved Caspase-3 and PARP compared with that in the negative control group in Tca8113 and SCC15 cells, and down-regulation of AEG-1 increased levels of cleaved Caspase-3 and PARP compared with that in the negative control group in SCC9 and CAL-27 cells. Taken together, these results indicate that AEG-1 reduced OSCC cell apoptosis.

### AEG-1 activates NF-κB and p38 signalling in OSCC cells

The NF-κB pathway is one of the key molecular pathways regulating cell survival^15^, and studies have suggested that AEG-1 induces NF-κB activation^19^. Therefore, we investigated whether AEG-1 modulates the p38 and NF-κB pathway in OSCC cells. Our western blotting results showed that down-regulation of AEG-1 in SCC-9 and CAL-27 cells decreased levels of p-p38, p-p65 and NF-κB p65 and that up-regulation of AEG-1 Tca8113 and SCC15 cells increased these levels (Fig. 6E,F). These data show that AEG-1 induced p38 and NF-κB activation in OSCC cells.

### Up-regulation of AEG-1 promotes tumour growth and EMT in the SCC15 xenograft model

**Figure 7:**
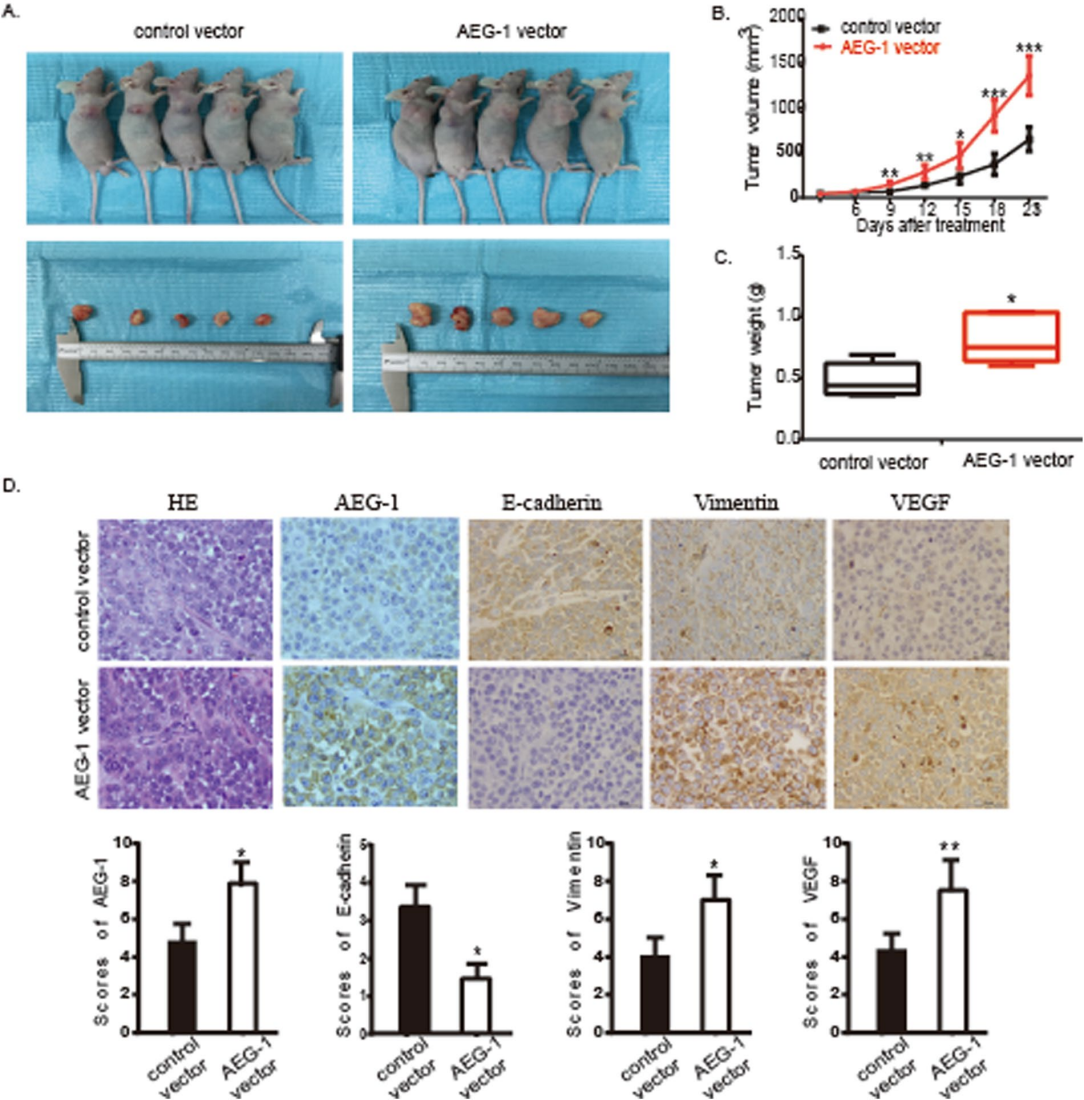
AEG-1 overexpression promoted tumour growth in a xenograft model. (**A**) The image of xenograft tumours established using control or AEG-1 transfected SCC15 cells. (**B**) The tumour growth curve indicated that AEG-1 up-regulation significantly promoted SCC15 xenograft tumour growth. (**C**) The average tumour weight of the AEG-1 overexpression group was increased compared to that of the sh-control group. (**D**) IHC staining showed expression of AEG-1, E-cadherin, vimentin and VEGF in the AEG-1 transfected SCC15 xenograft tumours (original magnification: 200×). *Indicates *P* < 0.05, **indicates *P* < 0.01, ***indicates *P* < 0.001 by *t*-tests. (SD, standard deviation).

To further verify the role of AEG-1 and to determine the therapeutic potential of targeting AEG-1 in OSCC, we established a xenograft tumour model using SCC15 cell lines. The tumour weight and growth curve suggested that AEG-1 increased tumour growth compared with that of the negative control (*P* < 0.05, Fig. 7A–C). The EMT markers E-cadherin and vimentin were suppressed and increased, respectively, tumours established by AEG-1-overexpressing SCC15 cells (*P* < 0.05, Fig. 7D). In addition, angiogenesis-related VEGF markers were increased in AEG-1-overexpressing tumours compared with in negative controls (*P* < 0.01, Fig. 7D). Taken together, these data indicate that AEG-1 overexpression dramatically promoted EMT and invasion of OSCC cells *in vivo* and that AEG-1 may serve as a therapeutic target for OSCC treatment.

## Discussion

As an intracellular hub molecule that regulates both NF-κB and mitogen-activated protein kinase (MAPK) signalling pathways, AEG-1 plays an important role in cell survival, promotion of metastasis and development of drug resistance^20^. AEG-1 is an oncoprotein that acts to modulate cell proliferation, angiogenesis, apoptosis, invasion and metastasis in many human malignancies^19,21,22^. Our group previously found a positive association between AEG-1 overexpression and distant metastasis, angiogenesis and survival^17^, lending further credence to the idea that AEG-1 is a clinically relevant promoter of OSCC progression and metastasis.

In the present study, we found AEG-1 to be aberrantly expressed in OSCC cells and that its up-regulation or knockdown significantly increased or inhibited, respectively, OSCC cell motility. It is well established that AEG-1 promotes tumour progression and metastasis in several malignancies, including liver carcinoma^23,24^ and osteosarcoma^25^, though its ability to enhance malignant phenotypes in OSCC remains poorly defined. One novel finding in our study is that AEG-1 expression is closely associated with that of vimentin and E-cadherin, two key EMT-related molecules. EMT entails acquisition of mesenchymal characteristics by epithelial cells, such as a vimentin-cytoskeleton and an elongated fibroblast-like morphology, as well as capacity for invasion and metastasis^26–28^.

In recent years, many studies have established a positive correlation between AEG-1 expression and EMT status in cancer progression, including in lung cancer^29^, cardiac myxoma^30^, glioblastoma^31^ and tongue squamous cell carcinoma (TSCC)^32^. In our study, we found increased expression of vimentin and decreased expression of E-cadherin in OSCC cell lines stably overexpressing AEG-1, which suggests that AEG-1 promotes EMT. In contrast, inhibition of AEG-1 expression caused increased expression of E-cadherin and decreased expression of vimentin, suggesting suppression or reversal of the EMT process. These results support the hypothesis that AEG-1 contributes to EMT and thus promotes cell invasion and metastasis in OSCC. Moreover, using a xenograft model, we also revealed that AEG-1 overexpression in implanted OSCC cells substantially increases tumour growth and angiogenesis and promotes the EMT process and invasion *in vivo*.

The role of NF-κB as a master transcriptional factor that can promote cell survival, increase therapeutic resistance and enhance the metastatic ability of cancer cells has been well documented^33,34^. Indeed, AEG-1 is an oncoprotein that acts to modulate various cell biological functions in many human malignancies. Our study examined whether AEG-1 is involved in the transmission of NF-κB signalling, and western blotting showed that AEG-1 induces p38 and NF-κB activation in OSCC cells. This discovery shows that AEG-1 promotes biological functions and molecular mechanisms in OSCC cells.

Both our data and that of Xia *et al*. clarified that AEG-1 is highly expressed in OSCC cell lines and tissue specimens. Xia *et al*. reported that AEG-1 expression is associated with histological differentiation, clinical stage, tumour size, lymph node metastasis and poor prognosis, suggesting that AEG-1 might serve as a prognostic predictive marker in OSCC. Our current study confirmed that regulating AEG-1 expression alters cell proliferation and invasion in OSCC. Taken together, AEG-1 plays an important role in OSCC cell carcinogenesis and may represent a potential therapeutic target.

In summary, we demonstrated that AEG-1 acts as an important oncogene in OSCC and that its expression is frequently up-regulated in human tumour samples and cancer cell lines. Functionally, AEG-1 promotes cell proliferation, angiogenesis, the cell cycle, drug tolerance, invasion and metastasis and inhibits apoptosis in OSCC cells. Upon further investigation into the molecular mechanism involved, we found that AEG-1 promotes tumour biological behaviour by inducing EMT and activates p38 and NF-κB signalling. Identifying dysregulated AEG-1 expression in OSCC cells will help to better our understanding of tumour progression in OSCC, identify candidate biomarkers for OSCC prognosis, and guide the development of therapeutic targets for oral cancer.

## Materials and Methods

### Cell culture and tissue specimens

Tissues from OSCC patients and normal specimens were obtained from patients immediately after surgical resection. The samples were flash frozen in liquid nitrogen and stored at −80 °C until use. All specimens were randomly selected from the Second Affiliated Hospital of Harbin Medical University, China. The Hacat cells and human OSCC cell lines Tca8113 and SCC4 were stored in our laboratory (Laboratory of Medical Genetics, Harbin Medical University); CAL-27 cells were kindly provided by Dr. Hongchen Sun (School of Stomatology, Jilin University), HN-6 and SCC9 cells by Dr. Chao Liu (Nanjing Medical University), and SCC15 cells by Dr. Xiaozhi Liu (Department of Neurosurgery, the Fifth Central Hospital of Tianjin). The human OSCC cell line Tca8113 was cultured in RPMI-1640 medium (Lonza). Hacat, SCC4, SCC15 and CAL-27 cells were grown in Dulbecco’s modified Eagle’s medium (DMEM, Lonza, Walkersville, MD, USA). The human OSCC cell lines HN-6 and SCC9 were cultured in DMEM/F12 (1:1) medium (Lonza). All cells were supplemented with 10% foetal bovine serum (FBS) (PAA Laboratories GmbH, Pasching, Australia) and cultured at 37 °C in a humidified 5% CO_2_ atmosphere.

### Lentiviral-mediated AEG-1 overexpression

A human AEG-1 lentiviral construct was generated by inserting the full-length human AEG-1 cDNA into the lentiviral vector Ubi-MCS-3FLAG-SV40-EGFP-IRES-puromycin (GeneChem, Shanghai, China). The human AEG-1 lentiviral expression plasmid or the GV358 control vector was co-transfected into 293T cells using Lenti-Pac HIV Packaging Mix (GeneChem, Shanghai, China). The lentivirus-containing supernatant was harvested at 48 h after transfection. To establish stable AEG-1-overexpressing cell lines, Tca8113 and SCC15 cells were transduced with lentiviral supernatant in the presence of 5 μg/ml polybrene and selected with 0.3 μg/mL puromycin. Stable overexpressing AEG-1 cells were obtained after antibiotic selection for 3 weeks, and expression of AEG-1 was confirmed by immunoblotting analysis.

### RNA interference

Oligonucleotides containing the following targeting sequences were used to clone shRNA-encoding sequences into hU6-MCS-Ubiquitin-EGFP-IRES-puromycin lentiviral RNAi GV248 vector (GeneChem, Shanghai, China). The following target sequences of AEG-1 were selected: 5′-AAGTCAAATACCAAGCAAA-3′ (sh-AEG-1-1), 5′-ATGATGAATGGTCTGGGTT-3′ (sh-AEG-1-2). The sh-control was 5′-TTCTCCGAACGTGTCACGT-3′. After co-transfection of lentiviral packaging plasmids into 293T cells, and the lentivirus-containing supernatant was harvested after 48 h. SCC9 and CAL-27 cells were transduced with the lentiviral supernatant in the presence of 5 μg/ml polybrene and selected with 0.3 μg/mL puromycin. Stable sh-AEG-1 cells were obtained after antibiotic selection for 3 weeks, and expression of AEG-1 was confirmed by immunoblotting analysis. Of the three sequences, we then selected the target sequence with the best transfection effect for subsequent experiments.

### Immunoblotting analysis

Cells were harvested in logarithmic phase and lysed with radioimmunoprecipitation assay (RIPA) buffer (150 mM NaCl, 1% NP-40, 0.25% Na-deoxycholate, 1 mM ethylenediaminetetraacetic acid (EDTA), 50 mM Tris-HCl, pH 7.4). Total protein and nucleus protein were extracted. Proteins were separated by 10% sodium dodecyl sulphate polyacrylamide gel electrophoresis (SDS-PAGE) and transferred onto polyvinylidene difluoride (PVDF) membranes (Millipore, Billerica, MA, USA). The membranes were incubated with the primary antibody overnight at 4 °C, with a secondary antibody (Zhongshan Bio-Tech Co., Beijing, China) for 1 h at room temperature, and then scanned using an Odyssey infrared imaging system (LICOR, Lincoln, NE, USA). Primary antibodies against AEG-1, E-cadherin, N-cadherin, vimentin (Abcam, UK), VEGF, p-p38, p38, Caspase-3 and PARP (Cell Signaling Technology Inc., Danvers, MA, USA), c-Myc, NF-κB p-p65, NF-κB p65 (Santa Cruz Biotechnology, Inc, Santa Cruz, CA, USA), and GADPH (KangChen Biotech, Shanghai, China) were used for western blotting analysis.

### Cell proliferation and colony formation assays

Two thousand Tca8113, SCC15, SCC9 and CAL-27 cells were seeded in 96-well plates and assayed for proliferation with MTS using CellTiter 96® AQueous One Solution Cell Proliferation Assay Kit (Promega Corporation, Madison, WI, USA). The optical density (OD) value of each well was measured every 24 h for 7 days, and each experiment was performed in triplicate. Statistical significance was analysed using two-way analysis of variance (*ANOVA*) with multiple comparisons *via* Bonferroni correction for each day, where *indicates *P* < 0.05, **indicates *P* < 0.01, and ***indicates *P* < 0.001. For the colony formation assay, 0.8 × 10^3^ Tca8113, SCC15, SCC9 and CAL-27 cells were plated in 6-well plates. After a 14-day period, the cells were washed with phosphate-buffered saline (PBS), fixed with 4% paraformaldehyde (Santa Cruz Biotechnology, USA) for 15 min and stained with Giemsa. Colony formation images are pictured for each well, and the number of colonies was counted using ImageJ software. All experiments were performed in triplicate. Statistical significance was analysed using *t*-tests correction for multiple comparisons, where *indicates *P* < 0.05, **indicates *P* < 0.01, and ***indicates *P* < 0.001.

### Cell viability assay

CDDP (≥98% in purity) was purchased from Dalian Meilun Biotech Co., Ltd. (Dalian, People’s Republic of China) and dissolved in 0.1% DMSO to create a stock solution of 20 μg/ml. Cells were seeded into 96-well plates (5 × 10^3^ cells/100 ml media/well) overnight under standard culturing conditions and treated with different concentrations (0, 0.156, 0.312, 0.625, 1.25, 2.5, 5, 10, 20 μg/ml) of CDDP for 72 h. Upon completion of the respective treatment period, the cell culture was added to MTS using CellTiter 96® AQueous One Solution Cell Proliferation Assay Kit (Promega Corporation, Madison, WI, USA) at 20 μl medium/120 ml well. After incubation for 4 h at 37 °C, the colour intensity was measured at 492 nm, and % inhibition was calculated using the formula, inhibition (%) = (1 − ODtest/ODvehicle control) × 100. The experiment was repeated three times at same passage number for each cell line. The data of three independent experiments were analysed with two-way *ANOVA*, where *indicates *P* < 0.05, **indicates *P* < 0.01, and ***indicates *P* < 0.001.

### Cell cycle analysis

Transfected OSCC cells were cultured in corresponding media, harvested, washed with PBS and fixed in 70% cold alcohol overnight at 4 °C. The cells were then washed and suspended in PBS and incubated with 100 μg/ml RNase and 40 mg/ml propidium iodide (PI) for 30 min at 4 °C. The cell cycle was analysed using a FACSCalibur flow cytometer (BD, USA). The distribution of cells in different phases of the cell cycle, such as G_0_/G_1_, S and G_2_/M, was estimated using ModFit LT software.

### Wound-healing assay

Cells were seeded in 6-well plates to confluence, and the cell monolayer was scraped in three straight lines with a 200-μl pipette tip to create ‘scratches’. Cell debris was removed by PBS, and fresh medium was added. Photographs of the Tca8113 cell wound area were taken at 0, 24 and 48 h; SCC15, SCC9 and CAL-27 cells were photographed at 0, 12 and 24 h after scratching. The experiments were performed in triplicate.

### Cell invasion assay

The cell invasion assay was performed using Transwell membranes coated with Matrigel (BD Biosciences, USA). Invasion chambers were rehydrated with 0.5 ml medium added to the upper chambers for 2 h at 37 °C. After rehydration, the medium was carefully removed, and 5 × 10^4^ transfected cells were added to the upper chambers in triplicate. The lower chamber was filled with 15% FBS. After 48 h incubation at 37 °C, cells remaining in the upper chamber were removed with cotton swabs; invading cells were fixed with 4% paraformaldehyde (Santa Cruz Biotechnology, USA), stained with Giemsa staining, and counted under a light microscope. Statistical significance was determined using *t*-tests, where *indicates *P* < 0.05, **indicates *P* < 0.01, and ***indicates *P* < 0.001.

### SCC15 xenograft tumour model

All animal protocols were approved by the Second Affiliated Hospital of Harbin Medical University Animal Care and Use Committee. Male, SPF grade Nu/Nu nude mice at 4 weeks of age (Slaccas, China; n = 6 for each group) were subcutaneously implanted with 5 × 10^6^ SCC15 transfected cells as previously described. The tumour volume was measured with a calliper every 3 days using a formula (volume = long diameter × short diameter^2^/2). After 3 weeks, the mice were euthanized and weighed, and the xenograft tumours were removed for formalin fixation and preparation of paraffin-embedded sections.

### Immunohistochemistry (IHC)

For IHC staining, paraffin-embedded tumour slides were deparaffinised, rehydrated and incubated with primary antibodies overnight at 4 °C. Primary antibodies against AEG-1, E-cadherin, vimentin and VEGF (Abcam, UK) were used for detection. The slides were then incubated with a biotin-labelled secondary antibody (Zhongshan Bio Corp., China) for 1 h at room temperature and then with diaminobenzidine (Zhongshan Bio Corp., China). The results were assessed by measuring both the staining intensity and the number of positive cells. The intensity of a positive reaction was scored as follows: 0 = negative, 1 = weak, 2 = moderate, and 3 = intense staining. Staining was also scored on a scale of 0–3 according to the percentage of cells involved, where 0 = 0–5%; 1 = 6–25%; 2 = 26–50%; and 3 = 51–100% positive cells. The scores for the intensity and the percentage of positive cells were multiplied to calculate a weighted score for each case. A score of 0–3 was defined as low expression (−) and scores of 4–9 as high expression (+).

### Statistical analysis

Data are expressed as the mean ± SD. Statistical analysis was performed using *t*-tests and *ANOVA* using GraphPad software. Statistical significance was determined at *P* < 0.05.

### Ethics statement

The methods used in this study were carried out in accordance with the approved guidelines of the Second Affiliated Hospital of Harbin Medical University Medical Ethnic Committee. All subjects provided written informed consent for participation in this study and for review of their medical records, and all subjects provided a sample of tumour tissue. We clarified that all the procedures using human tissues, including tissue sample collection, protein extraction and western blotting, were carried out in accordance with the Human Tissues Use Guidelines of the Second Affiliated Hospital of Harbin Medical University Medical Ethnic Committee. All experimental animal protocols were carried out in accordance with Experimental Animal regulations of the Second Affiliated Hospital of Harbin Medical University Animal Care and Use Committee.

## Electronic supplementary material

Supplementary Information
