# Supplementary material for: Astrocyte elevated gene-1 promotes tumour growth and invasion by inducing EMT in oral squamous cell carcinoma
Source: Sci Rep. 2017 Nov 13;7:15447. doi: 10.1038/s41598-017-15805-8 (PMC5684276; doi:10.1038/s41598-017-15805-8)
Supplement: Supplementary file 1 — Supplementary Information [file 41598_2017_15805_MOESM1_ESM.pdf]

Astrocyte elevated gene-1 promotes tumour growth and invasion by inducing EMT in oral squamous cell carcinoma

Yan Wang[1], Ting Wang[1], Yunduan Sun[2], Wenjing Sun[3], Xiumei Wang[1]*

1 Department of Dentistry, the Second Affiliated Hospital of Harbin Medical University, Harbin 150086, China.

2 Department of Ophthalmology, the First Affiliated Hospital of Harbin Medical University, Harbin 150001, China.

3 Laboratory of Medical Genetics, Harbin MedicalUniversity, Harbin 150081, China.

Corresponding author: Prof. Xiumei Wang, Department of Dentistry, the Second Affiliated Hospital of Harbin Medical University, 246 Xuefu Road, Nangang District, 150086 Harbin, China.

E-mail address: hrbwangmei@163.com (X. Wang).

Telephone: +86-451-86605544

Ethics statement

The methods used in this study were carried out in accordance with the approved guidelines of the Second Affiliated Hospital of Harbin Medical University Medical Ethnic Committee. All subjects provided written informed consent for participation in this study and for review of their medical records, and all subjects provided a sample of tumour tissue. We clarified that all the procedures using the human tissues, including colleting the tissue samples, protein extraction and western blot, were carried out in accordance with the Human Tissues Use Guidelines of the Second Affiliated Hospital of Harbin Medical University Medical Ethnic Committee. All experimental animal protocols were carried out in accordance with Experimental Animal regulations of the Second Affiliated Hospital of Harbin Medical University Animal Care and Use Committee.

Acknowledgments

This work was supported by the Science and Technology Innovation Talent Research Special Fund Project of Harbin Municipal of China (2016RAXYJ094 to X.M.W.) and Leading Talent Echelon Fund for Reserve Leaders of Heilongjiang Province (grant to X.M.W.) .

Author Contributions

Y.W. and X.M.W. planned all experiments. T.W. and Y.W. collected all tumour samples. Y.W. performed most of the experiments and all the *in vitro* experiments and collected and analysed data. T.W. mainly performed the *in vivo* experiments. Y.W. drafted the manuscript. X.M.W., W.J.S. and Y.D.S. helped with the experiments and reviewed the manuscript. All authors approved the final version of the manuscript for submission.

Additional Information

Supplementary information accompanies this paper at http: //www. Nature.com/srep

Competing financial interests The authors declare no competing financial interests.

This work is licensed under a Creative Commons Attribution 4.0 International License. The images or other third party material in this article are included in the article's Creative Commons license, unless indicated otherwise in the credit line; if the material is not included

under the Creative Commons license, users will need to obtain permission from the license holder to reproduce the material. To view a copy of this license, visit http://creativecommons.org/licenses/by/4.0/

Table 1. IC50 dosage of the extract for the respective OSCC-treated cell line.

Cells	Tca8113		SCC15		SCC9		CAL-27	
	Control vector	AEG-1 vector	Control vector	AEG-1 vector	sh-control	sh-AEG-1	sh-control	sh-AEG-1
IC50 (μg/ml)	2.2	7.3	5.9	8.6	11.3	6.23	11.1	8.8
